# Insulin Receptor Isoforms and Insulin Growth Factor-like Receptors: Implications in Cell Signaling, Carcinogenesis, and Chemoresistance

**DOI:** 10.3390/ijms241915006

**Published:** 2023-10-09

**Authors:** Mariam Ahmed Galal, Samhar Samer Alouch, Buthainah Saad Alsultan, Huda Dahman, Nouf Abdullah Alyabis, Sarah Ammar Alammar, Ahmad Aljada

**Affiliations:** 1 Department of Biochemistry and Molecular Medicine, College of Medicine, Alfaisal University, Riyadh 11533, Saudi Arabia; 2 Department of Translational Health Sciences, Bristol Medical School, University of Bristol, Bristol BS8 1QU, UK

**Keywords:** insulin receptor isoforms, insulin growth factor-like receptors, chemoresistance, insulin signal transduction, IGF signal transduction

## Abstract

This comprehensive review thoroughly explores the intricate involvement of insulin receptor (IR) isoforms and insulin-like growth factor receptors (IGFRs) in the context of the insulin and insulin-like growth factor (IGF) signaling (IIS) pathway. This elaborate system encompasses ligands, receptors, and binding proteins, giving rise to a wide array of functions, including aspects such as carcinogenesis and chemoresistance. Detailed genetic analysis of IR and IGFR structures highlights their distinct isoforms, which arise from alternative splicing and exhibit diverse affinities for ligands. Notably, the overexpression of the IR-A isoform is linked to cancer stemness, tumor development, and resistance to targeted therapies. Similarly, elevated IGFR expression accelerates tumor progression and fosters chemoresistance. The review underscores the intricate interplay between IRs and IGFRs, contributing to resistance against anti-IGFR drugs. Consequently, the dual targeting of both receptors could present a more effective strategy for surmounting chemoresistance. To conclude, this review brings to light the pivotal roles played by IRs and IGFRs in cellular signaling, carcinogenesis, and therapy resistance. By precisely modulating these receptors and their complex signaling pathways, the potential emerges for developing enhanced anti-cancer interventions, ultimately leading to improved patient outcomes.

## 1. Introduction

Cancer is a major cause of mortality worldwide accounting for the death of nearly 10 million cases and 19.3 million new cases in 2020, showing an increase of 15% and 10.3% compared to 2015, respectively [1]. The formidable challenge of chemoresistance further complicates cancer treatment, leading to relapses and metastases and accounting for a staggering 90% of cancer-related fatalities [2,3,4]. Insulin receptors (IRs) and insulin-like growth factor receptors (IGFRs) have been reportedly linked to carcinogenesis and chemoresistance. While IRs serve as key mediators of insulin and its intercellular pathways, influencing cellular metabolism and mitosis, IGFRs play vital roles in cellular proliferation and survival. This comprehensive review investigates the formation, structure, and regulation of IR isoforms and IGFRs in the human body, exploring their activation and downstream signaling pathways and examining their significant involvement in both carcinogenesis and chemoresistance.

## 2. IRand IGFR Genes and Structures

### 2.1. IR

#### 2.1.1. INSR Gene and IR Isoform Formation

The human IR is encoded by the *INSR* gene, which comprises 22 exons spanning 120 kb and is located on chromosome 19p13.2. The first 11 exons encode the alpha subunit, while the remaining 11 exons encode the beta subunit of the receptor. *INSR* cDNA cloning was first reported in 1985 [5,6], and the predicted transcripts showed different lengths due to the alternative splicing of exon 11, which spans 36 bp. As a result, two IR isoforms are produced: IR-A (1370 amino acids) and IR-B (1382 amino acids) [7]. Notably, the 12-amino acid sequence (residues 745–756) derived from exon 11 is present in the IR-B isoform but absent in the IR-A isoform [8,9]. 

**Figure 1:**
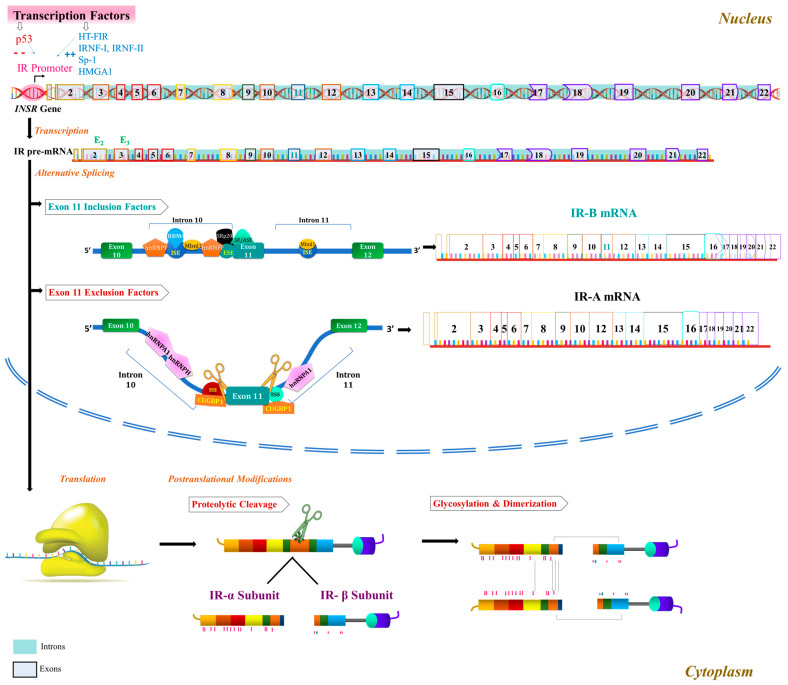
The formation of IR isoforms: (1) Gene expression: *INSR* gene encoding IR is transcribed from DNA in the nucleus. (2) Pre-mRNA splicing involving introns’ removal and exclusion/inclusion of E11 to form mature mRNA molecules that encode either IR-A or IR-B isoforms; this process is regulated by specific splicing factors that bind to *cis*-acting elements in the pre-mRNA. (3) mRNA export from the nucleus to the cytoplasm through nuclear pores to serve as templates for protein synthesis. (4) mRNA Translation for IR synthesis. (5) Post-translational modifications (PTMs) in the endoplasmic reticulum and Golgi apparatus, including glycosylation, disulfide bond formation, and proteolytic cleavage. (6) Protein trafficking and secretion: mature IR isoforms are transported from the ER to the plasma membrane, where they are inserted and anchored by transmembrane domains. This process occurs through vesicular transport and fusion with the plasma membrane. Once inserted into the plasma membrane, IR isoforms are available for ligand binding and downstream signaling.

Regulation of IR expression involves various factors at the transcriptional and mRNA levels [10] (Figure 1). Several factors upregulate IR expression at the promoter level, such as the hepatocyte-specific transcription factor of the *IR* gene [11], IR nuclear factor I and II [12,13], and the transcription factor high-mobility group protein A_1_ (HMGA_1_) [14]. Conversely, p53 downregulates its expression [15]. MicroRNAs (miRNAs) also play a role in post-transcriptional regulation [16], with liver-specific miRNAs (miRNA-15b [17], miR-195 [18], miR-497 [19], and miR-103/107 [20] suppressing IR expression by targeting its 3′untranslated region (UTR). Additionally, let-7 miRNA family members, which are tumor suppressors, suppress IR expression [21]. 

The modulation of alternative splicing critically governs the expression of distinct IR isoforms. Within the breakpoint sequence of intron 10 and in exons 10, 11, and 12, essential regulatory elements have been identified, coordinating the splicing process. Notably, CUG-binding protein1 (CUGBP1) was the first known splicing regulator of exon 11 to promote IR-A isoform by binding two silencer sequences, one upstream of exon 11 at the 3′end of intron 10 and another in the middle of exon 11 [22]. Heterogeneous nuclear ribonucleoprotein family proteins (hnRNPs) have also emerged as significant splicing regulators involved in IR pre-mRNA splicing and mRNA export, stability, and translation [23]. Specifically, hnRNPF and hnRNPA1 exhibit antagonistic roles by binding to GA-rich intronic and exonic splicing regulatory elements, influencing the alternative splicing of exon 11. hnRNPF promotes IR-B expression by binding to both ends of intron 10, facilitating the inclusion of exon 11. On the other hand, hnRNPA1 enhances IR-A expression by binding to intron 10 and the 5′ splice site of intron 11, resulting in the exclusion of exon 11 [24]. Moreover, Muscleblind-like 1 (MBNL1), belonging to the muscleblind-like (MBNL) proteins, positively regulates the expression of the IR-B isoform. MBNL1 recognizes an intronic enhancer element within intron 11, thus promoting IR-B isoform expression [22] and counteracting the effects of CUGBP1 and hnRNPH [25].

Serine–arginine-rich (SR) proteins play a crucial role in the alternative splicing of mRNA by binding to exonic and intronic sites and interacting with small nuclear ribonucleoproteins [26]. Specifically, the splicing factors SRp_20_ and SF_2_/ASF exert their influence by promoting the formation of IR-B through their binding to the enhancer sequence at the 5′ end of exon 11, while concurrently antagonizing the actions of CUGBP1 [22]. Additionally, the RNA binding motif4 (RBM4) protein has been identified as a promoter of exon 11 inclusion, favoring IR-B expression by binding to GC-rich motifs [27]. Furthermore, Malakar et al. demonstrated the regulatory role of the Ras-MEK1-ERK pathway in this alternative splicing event, suggesting its potential impact on exon 11 inclusion and consequently influencing IR-B isoform levels [28]. 

#### 2.1.2. IR Structure

**Figure 2:**
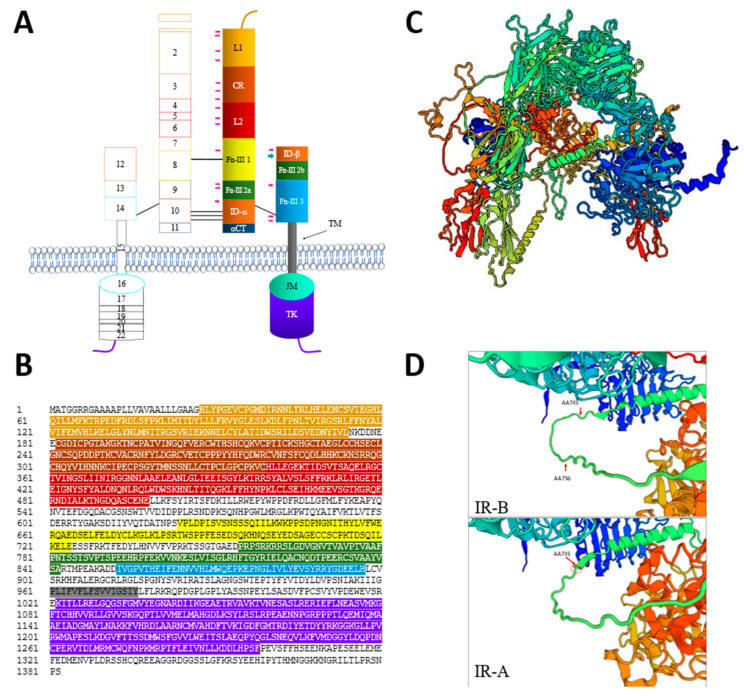
Structure of IR receptors. (**A**) Illustration showing different IR domains encoded by the 22 exons. IR has two main subunits: α and β. The α subunit contains 5 main domains, L1 (AA 28–174) CR (AA 182–339), and L2 (AA 340–497), and 2 Fibronectin subunits: FnIII-1 (residue 624–726) and FnIII-2 (757–842). The two α-subunits are linked by a disulfide bond between the two Cys 524 in the first FnIII domain. One to three of the triplet Cys at 682, 683, and 685 in the insert within the second FnIII domain are also involved in α-α disulfide bridges. There is a single disulfide bridge between α and β subunits between Cys 647 in the insert domain and Cys 872. The β subunit details are explained in the main text. Teal arrow shows 6 O-glycosylations, whereas the pink arrows imply the N-glycosylation. (**B**) IR-B amino acid sequence colored to identify each domain sequence presented in (**A**). (**C**) 3D structure of IR-B showing the Λ-shaped structure when no ligand is bound to it. (**D**) Structural differences between IR-A and IR-B. JM: Juxtamembrane, TK: Tyrosine Kinase. The 3D models were created using Swiss Model.

The IR is a transmembrane receptor with a molecular weight of (156 kDa), representing a classical example of the tyrosine kinase family [29]. It spans the cell’s plasma and was first characterized in 1971 when the specific binding of ^125^I-labeled insulin to the plasma membrane of adipose tissue and liver cells was detected [30]. Over the years, various methods have been employed to elucidate its structure, including insulin-agarose affinity chromatography [31], photoreactive insulin derivatives [32], and the use of bifunctional cross-linking agents such as disuccinimidyl suberate [33], with subsequent analysis performed using sodium dodecyl sulfate-polyacrylamide gel electrophoresis (SDS-PAGE). In 2006, the first 3D structures of the human IR ectodomain were reported [34,35]. The *INSR*-encoded IR chain is cleaved by the enzyme furin, generating α subunits (135 kDa) and β subunits (95 kDa) (Figure 2). Following dimerization with another α- β pair, the subunits undergo proteolytic cleavage, and the two dimers become covalently linked by disulfide bonds, forming a 450 kDa protein, disregarding post-translational glycosylation [29,36]. 

The α subunit of IR comprises the N-terminal portion of the extracellular domain, characterized by rich leucine repeat L1 and L2 separated by a highly marked cysteine region (CR). It also includes one of the fibronectin type III domains (FnIII-1) and two amino acids (FnIII-2). Ligands can bind to the IR receptor either at the cysteine-rich domain in the extracellular region or at the 16-aminoacid residue CT peptide [37,38]. In contrast, the β subunit contains a C-terminal ectodomain (residues 763–956), a transmembrane helix domain (residues 957–979), and a cytoplasmic domain consisting of juxtamembrane segment and intercellular tyrosine kinases, consecutively (residues 980–1382) [39,40]. The rest of the FnIII-2 is located together with (FnIII-3) on the β subunit. Notably, a relatively disordered insert domain (ID) containing the α/β furin cleavage site is located within the canonical CC′ loop of FnIII-2. A peptide segment from the C-terminal region of the ID α-chain component (IDα) is known as αCT. The length of the αCT segment varies in the IR-A and IR-B, depending on whether exon 11 is included or excluded in the IR. Of significance, the α subunit, along with 149 residues of the β subunit, constitutes the ectodomain of the IR receptor, displayed as Λ-shaped structure. In the ligand-unbound state, the “Λ” conformation of IR is formed by the L1-CR-L2 module of one monomer packing against the FnIII-1, -2, and -3 modules of the alternate monomer (Figure 2C) [39]. There are two distinct classes of disulfide bonds (S-S) in the IR structure. Class I disulfide bonds link the α-subunits within the homodimer, while class II disulfide bonds form between the α and β monomers. Intermonomer disulfide bonds occur between the FnIII-1 domains and between the segments of the insert domain α chain component (IDα). Additionally, within each monomer, a disulfide bond links IDα to the FnIII-3 domain, which is part of the β-chain. The IR exhibits an extensive glycosylation pattern, with nineteen N-glycans and six O-glycans distributed across the α-subunit and the extracellular part of the β-subunit. This glycosylation plays a crucial role in protein folding, processing, and membrane translocation, facilitating the proper functioning of the receptor [41,42]. Apart from its pivotal role in maintaining glucose homeostasis, the IR can also stimulate cell growth through its cytoplasmic carboxy-terminal domain within the intracellular β-subunit. This domain moderates the mitogenic actions of the receptor, contributing to its diverse cellular functions [43,44]. 

#### 2.1.3. Characteristics of IR Isoforms

As indicated above, there exist two isoforms of IRs: IR-B and IR-A. These isoforms are distinguished by the inclusion or exclusion of exon 11, resulting in length differences between the two isoforms. Additionally, these two transcript variants vary in their affinity for ligand binding, their distribution throughout the body, and their functions (Table 1). IR-A exhibits higher binding affinity for insulin binding [45,46]. However, it has a significantly stronger affinity for IGFs, particularly for IGF-II, compared to IR-B [47]. Moreover, IR-A has a greater internalization and recycling rate [48]. 

IRs primarily exist in target organs like the liver, adipose tissue, and skeletal muscle since insulin serves as their major ligand. IRs are also present in various other tissues, including the brain, heart, kidney, pulmonary alveoli, pancreatic acini, placenta vascular endothelium, monocytes, granulocytes, erythrocytes, and fibroblasts [59]. When examining the distribution of IR isoforms, it becomes apparent that it varies across different tissues. For instance, IR-A is predominantly expressed in fetal tissues, the brain, ovaries, testes, and the spleen, whereas IR-B expression is most abundant in the liver as well as pancreatic beta cells and muscle, adipose tissue, and kidney cells [8,60,61]. However, some tissues do express both isoforms in relatively equal proportions, such as in pancreatic islets [62] and skeletal muscle [8]. Importantly, the distribution of IR isoforms is not only specific to certain tissues but also varies within different cell types. For example, in the liver, hepatocytes predominantly express IR-B (approximately 90%), whereas endothelial cells in the liver mainly express IR-A [63,64]. Similarly, while IR-A is the primary isoform expressed in the brain, IR-B expression is more prominent in human astrocytes [65]. 

The aforementioned differences lead to a preference for IR-B in metabolic and differentiating signaling pathways. IR-B serves as the primary regulator of glucose homeostasis, initiating stronger kinase activation and playing a crucial role in metabolic effects [46,66]. On the other hand, IR-A predominantly promotes cell growth, proliferation, and survival, particularly contributing to cellular growth during fetal development. Furthermore, IR-A receptors demonstrate greater efficiency in mediating receptor endocytosis and insulin degradation. Notably, in hepatocytes, IR-A is more effective than IR-B in increasing glycogen synthesis, glycogen synthase activity, and glycogen storage [67]. The biological effects of ligand binding are also remarkably influenced by the isoform involved. For instance, in 32D cells, IGF-II binding to IR-A induced mitogenic and antiapoptotic signals while binding to IR-B stimulated differentiation signals [68]. In mouse fibroblasts lacking IGF-1R and transfected with the IR-A, insulin binding leads to metabolic effects, while IGF-II triggers mitogenic effects [69]. 

### 2.2. IGFR

#### 2.2.1. Type 1 Insulin-Like Growth Factor Receptor (IGF-1R) 

The *IGF-1R* gene located on chromosome 15q26.3 encodes both the α and β subunits of the IGF-1R. After the translation of IGF-1R mRNA, the pro-IGF-1R protein product (210 kDa) undergoes endoproteolytic cleavage by the pro-protein convertases such as furin and pro-protein convertase 5. This cleavage results in the generation of the α chain (706 amino acids) and β chain (627 amino acids) [70]. The mature IGF-1R forms a heterotetramer consisting of two α chains and two β chains [71]. Notably, both IGF-1R (154 KDa) and IR share structural similarities as heterodimeric tyrosine kinase receptors, each composed of two α-subunits and two β-subunits [72,73].

The α chain of the IGF-1R receptor contains a fused Fibronectin III domain, including FnIII1 (491–609) and FnIII2 (610–708), and the first two amino acids of FnIII3 (735–828). On the other hand, the β chain begins with the rest of FnIII3, followed by FnIII4 (834–927) and a long protein kinase region (999–1274). Similar to the IR β subunit, the IGF-1R β chain consists of an extracellular domain (741–935), a transmembrane helical domain (936–959), and an intercellular cytoplasmic domain (960–1367). Comparably, IGF-1R receptors are homologous to IRs by 45–65% in the ligand-binding domains and by 65–85% in the tyrosine kinase and substrate recruitment domains [72,74].

Upon insulin binding to its receptor IR, the receptor becomes activated and plays a crucial role in modulating glucose homeostasis, glycogen synthesis, protein synthesis, and lipogenesis, among other processes [75]. Similarly, IGF-1R gets activated by its special ligands IGF-1 (195 amino acids) and IGF-2 (180 amino acids), primarily produced by the liver and to a lesser extent by extrahepatic sites. Ligand binding induces a conformational change in the receptor, transmitting a signal that plays a significant role in normal human growth [76] (Table 1).

#### 2.2.2. Type 2 Insulin-Like Growth Factor Receptor (IGF-2R)

In contrast to IR and IGF-1R, IGF-2R (2491 AA ≈ 250 kDa) is a mannose 6-phosphate (MP6) receptor that lacks tyrosine activity. IGF-2R can be divided into three regions: a large extracellular region (2304 amino acids) facing the lumen, known as the lumenal region, a helical region (23 amino acids), and a cytoplasmic region. The lumenal region comprises a 40-residue amino acid signal sequence and 15 homologous repeat domains, each ranging between 124 and 192 amino acids [77]. 

IGF-2R is a multifunction transmembrane glycoprotein that binds both IGF-2 and MP6-marked lysosomal enzymes at various domains [78,79,80] (Table 1). Upon binding, IGF-2R mediates several processes, including the transportation of newly synthesized lysosomal enzymes from trans-Golgi to the endosome [79], suppression of IGF-2 proliferative actions [81], and cleavage of growth factors, yielding an effective growth inhibitory role for almost all cell types [82]. Indeed, IGF-2R is responsible for transporting IGF-2 to the cell for degradation, thereby regulating its circulating and tissue amount [77]. The receptor is expressed widely in fetal and neonatal tissues, contributing to the regulation of cell growth. However, its expression decreases after childbirth [83]. Importantly, IGF-2R expression can be observed in adipocytes originating from both subcutaneous and visceral adipose tissues [84]. 

In addition, IGF-2R effectively clears apoptotic cells, thus maintaining the stability of tissue environments [85], and activates transforming growth factor beta (TGFβ) [86], which is involved in immune regulation, extracellular matrix synthesis, as well as the proliferation, differentiation, and development regulation of various types of cells [87]. Hence, there is a prevailing notion that IGF-2R plays a role in suppressing tumors, and any changes in its expression, as elaborated in the subsequent section, could contribute to carcinogenesis.

### 2.3. Hybrid Receptor (HR)

In many cells, both isoforms of the IR are co-expressed, enabling them to form homodimers, resulting in IR-A and IR-B, or heterodimers, giving rise to IR-A/IR-B hybrid receptors [10]. These hybrid receptors are formed randomly within the cells [57,88]. Similarly, when IR and IGF-1R are co-expressed, IR isoforms can heterodimerize with the IGF-1R receptor where the hemi-receptor of α- and β-IR subunits combine with the α- and β-IGF-1R hemi-receptor subunits [57]. Because of the existence of two IR isoforms, the formed IR/IGF-1R hybrids can be either IR-A/IGF-1R or IR-B/IGF-1R. This heterodimerization occurs efficiently as homodimerization due to the homology between the two receptors. Consequently, the proportion of hybrid receptors depends on the mole fraction of each individual receptor [89]. Therefore, the less abundant receptors (IR or IGFR) in any cell type are commonly found in hybrids rather than in classical homodimers.

The IR/IGF-1R hybrid receptor possesses two ligand-binding sites: the first comprises the L1 domain of the IR and the α CT segment of the IGF-1R, while the second involves the L1 domain of the IGF-1R and the α CT segment of the IR [90]. Remarkably, the hybrid receptor binds IGF-2 with similar affinity to IGF-2 receptors, whereas the affinity of the hybrid receptor to insulin is lower than the IRs [91,92]. When comparing IR-A/IGF-1R hybrids to IR-B/IGFR hybrids, the former exhibits significantly higher ligand affinity [52]. However, the specific details concerning the signaling pathways and the role of hybrid receptors in carcinogenesis extend beyond the scope of this review.

## 3. Receptor Activation and Signaling Pathway

As previously mentioned, IR and IGF-1R exhibit a significant level of similarity in their molecular structure. Upon binding with their respective ligands, these receptors activate common intracellular mediators that play crucial roles in regulating cell metabolism, proliferation, and survival [93,94,95]. Typically, IRs primarily mediate anabolic effects, while IGF-1R predominantly modulates antiapoptotic, mitogenic, and transforming effects. However, it is now evident that IRs also have mitogenic and transforming functions. This segment will examine the receptor activation mechanisms, the pertinent signaling routes, and the ensuing downstream ramifications.

### 3.1. Activation of Receptor Tyrosine Kinase (RTK) Superfamily

The activation of RTKs occurs when a ligand binds to their extracellular domain, leading to the activation of their intracellular tyrosine kinase domain. Unlike most RTK receptors, the IR superfamily, including the IR and IGF-1R, exists as a covalent disulfide-linked dimer in the absence of a ligand. Upon ligand binding to the dimerized RTK, the kinase domain undergoes activation through the transphosphorylation of β-subunits, resulting in the specific tyrosine amino acid residue within the intracellular domain becoming phosphorylated. The phosphorylated residues then serve as binding sites for signaling partner proteins that possess homology 2 (SH2) domains. Subsequently, these partner proteins get phosphorylated by the kinase, initiating an intracellular signal transduction cascade [39,96,97].

#### 3.1.1. Proposed IR Activation Mechanisms

**Figure 3:**
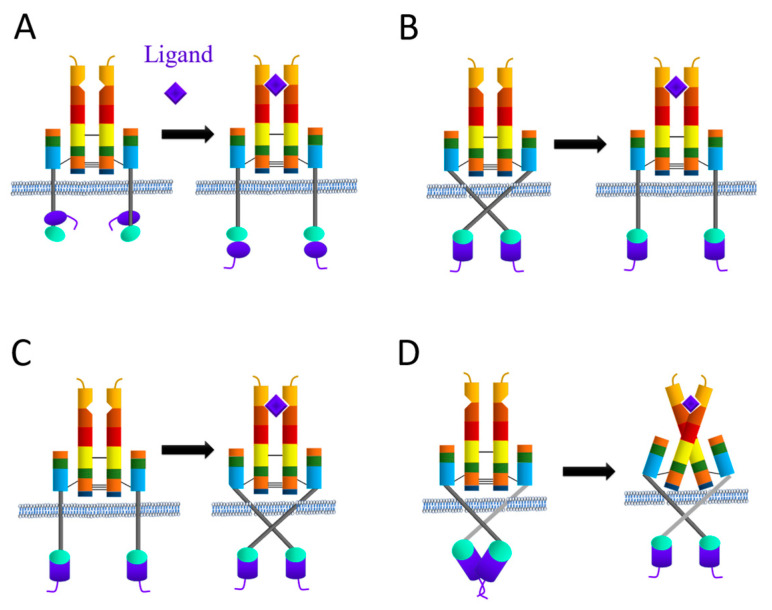
Hypothetical mechanisms of IR receptor activation upon ligand binding as proposed in the literature. (**A**) Ward et al. [40]; (**B**) Lee et al. [99]; (**C**) Kavran et al. [100]; (**D**) Maruyama et al. [97].

When no ligand is bound to the ectodomain, the IR exists as an apo monomeric form. Although recent research has provided detailed structures of the extracellular and intracellular domains of the insulin and IGF-1 receptors, a complete structure of the liganded and unliganded receptors remains elusive. As a result, the specific mechanism by which extracellular ligand binding activates the intracellular kinase domain is not yet fully understood. However, various hypotheses have been proposed in the literature (Figure 3). One hypothesis, put forward by Ward et al. [40] suggests that ligand-induced conformational changes lead to the descent of the kinase domains, akin to a yo-yo, from their constrained position near the membrane, where they are partially surrounded by the juxtamembrane region. This release allows the kinases to approach each other and undergo transphosphorylation. The model finds partial support from the 3D structure of a juxtamembrane-inhibited kinase domain [98]. On the other hand, Lee et al. hypothesized that ligand binding (insulin) causes the separation of the transmembrane domains within the receptor, which were held together in the inactive form of the receptor [99]. However, no structural studies have yet been conducted to support this hypothesis. Another hypothesis, proposed by Kavran et al. [100], suggests that the activation mechanism involves separation of the transmembrane domains, enforced by the unliganded extracellular domain, which maintains the receptor in its inactive state. When the ligand binds, it removes the inhibition by disturbing the L1-FnIII2′-3′ interaction. According to this view, ligand binding removes the inhibition rather than directly stimulating the activity of the phosphorylated receptor, which contradicts the mechanism speculated by Lee et al. A more recent mechanism proposed by Maruyama suggests that ligand binding to the extracellular domain stimulates a rotation of the transmembrane domains, followed by rearrangement and/or activation of the intracellular domain [97]. However, further research is needed to fully elucidate the exact activation process.

#### 3.1.2. Intracellular IR Signal Transduction Network

Upon ligand binding to the extracellular domain, the IR-tyrosine kinase is activated through the triphosphorylation of its activation loop. The activated kinase then proceeds to phosphorylate specific tyrosine residues, creating binding sites for signaling protein partners containing SH2 or PTB (phosphotyrosine-binding) domains. Unlike other RTKs, the IR and IGF-1R do not directly bind to signaling proteins. Instead, a family of large docking proteins called insulin receptor substrates (IRSs) [94,101,102] and adapter proteins like Shc, Gab1, Cbl, APS (associate protein substrate), and members of the signal regulatory protein family [103] associate with phosphorylated tyrosine residues in the juxtamembrane domain (Figure 3). 

Six types of IRSs are identified as IRS1 through IRS-6, serving as scaffolds to organize and mediate signaling complexes [102,104,105,106]. Although these IRSs have similar tyrosine phosphorylation motifs, they exhibit distinct functions in vivo. For instance, IRS-1 is crucial for myoblast differentiation and glucose metabolism in skeletal muscle [107,108]. IRS-1 knockout (KO) mice show growth retardation and impaired insulin action, though they maintain normal glucose tolerance [109]. Additionally, IRS-1 KO preadipocytes show defects in differentiation [110,111]. Conversely, IRS-2 KO mice display growth reduction in specific tissues such as neurons and islet cells and exhibit defective insulin signaling in the liver, potentially leading to diabetes when combined with beta cell loss [112]. IRS-2 KO preadipocytes differentiate normally but have impaired insulin-stimulated glucose transport [110,111]. In skeletal muscles, IRS-2 is important for lipid metabolism and ERK activation [107,108]. IRS-3 and IRS-4 have more restricted tissue distribution patterns. IRS-3 is highly abundant in various tissues in rodents, but it is a pseudogene in humans and thus does not produce any protein [113]. IRS-4 mRNA is present in the skeletal muscle, liver, heart, and kidney [114]. IRS-4 KO mice show minimal growth retardation and glucose intolerance [115]. IRS-5 and IRS-6 have limited tissue expression and act as relatively poor substrates for IR [116]. Insulin effects are primarily mediated through the interaction of IRS-1 and IRS-2 and Shc with the IR [117,118,119]. IRS proteins possess an N-terminal pleckstrin homology (PH) domain adjacent to the PTB domain, which facilitates their recruitment to the activated IR. The central and C-terminal parts of IRS proteins contain multiple potential phosphorylation sites that bind to signaling proteins with SH2 domains [39]. 

Insulin signaling involves two main pathways. The first pathway operates through the phosphatidylinositol 3-kinase (PI3K)/AKT (also known as the protein kinase B (PKB)) pathway, which generates the metabolic effects of insulin and is connected through IRSs. The second pathway involves the Raf/Ras/MEK/MAPK (mitogen-activated protein kinase, also known as extracellular signal regulated kinase (ERK)) pathway, which regulates gene expression and, along with the PI3K pathway, controls cell growth and cell differentiation [118]. 

#### 3.1.3. PI3K Signaling Pathway 

The regulatory subunit of PI3K has different isoforms encoded by three different genes. Pik3r1 encodes around 65–75% of all regulatory subunits, primarily in the form of p85α, along with the splice variants p55α and p50α. Pikr2 encodes p85β, accounting for 20% of the regulatory subunits. Pik3r3 encodes p55γ, structurally similar to p55α but expressed at lower levels in most tissues [116]. Activation of the PI3K pathway occurs when the p85 or p55 regulatory subunits of PI3K bind to IRS-1 and IRS-2, leading to the activation of the p110 catalytic subunit. This activation results in the phosphorylation of phosphatidylinositol 4,5-bisphosphate (PIP2) to form the lipid second messenger phosphatidylinositol (3,4,5)-triphosphate (PIP3). The formation of PIP3 activates three isoforms of AKT/PKB through an enzyme called phosphoinositide-dependent protein kinase 1 and 2 (PDK-1 and PDK-2). PDKs are activated by binding to inositol 1,4,5-trisphosphate (IP3) in the cell membrane [118,120,121]. 

There are four important downstream substrates of AKT/PKB. The first is the mammalian target of rapamycin (mTOR), a serine/threonine kinase involved in regulating protein synthesis. mTOR stimulates protein synthesis by phosphorylating eukaryotic translation initiation factor 4E binding protein 1 (4EBP1) and p70 ribosomal protein S6 kinase (p70S6K). When mTOR phosphorylates 4EBP1, it releases eIF-4E, which allows it to interact with eIF-4G, thereby activating cap-dependent mRNA translation [122]. The second substrate is glycogen synthase kinase 3 (GSK3), a serine/threonine protein kinase that regulates glycogen synthesis by inhibiting glycogen synthase. GSK3 is inhibited when phosphorylated by AKT/PKB [123]. The third substrate is the forkhead box-containing protein, O superfamily (FoxO), a transcription factor involved in the regulation of gluconeogenic and adipogenic genes. In the absence of insulin, FoxO translocates to the nucleus and induces the expression of genes such as PEPCK (phosphoenolpyruvate carboxykinase), a key enzyme in gluconeogenesis, and cyclin G2, an atypical cyclin that blocks the cell cycle. Insulin inhibits FoxO1 by phosphorylating it with AKT, leading to its sequestration in the cytoplasm. FoxO1 also plays a critical role in metabolism and longevity under the name daf16 [124]. The fourth one is AS160, an AKT substrate of 160kDa, which is involved in glucose transport [125]. 

#### 3.1.4. The MAPK-ERK Signaling Pathway 

The adaptor protein known as Grb2 (growth factor receptor-bound protein 2) binds to IRSs and Shc, forming a complex with the SOS (son of sevenless), a guanine nucleotide exchange factor that stimulates GDP/GTP exchange on the small G protein p21 [126]. This complex triggers the activation of a cascade of serine/threonine kinases (Raf/MEK/ERK1/ERK2). Upon activation, the serine/threonine kinase Raf phosphorylates the dual-specificity kinase MAPK (MEK1), which in turn phosphorylates ERK1/2. The phosphorylated ERK1/2 dissociates from MEK1 and translocates to the nucleus, where it phosphorylates several transcription factors. Additionally, it targets stress- and mitogen- activated protein kinases [10,127,128]. It is noteworthy to mention that RAS activation also activates the PI3k pathway by activating p110 independently of p85 [129].

#### 3.1.5. Intracellular Domain Pathway (LYKi)

In addition to the conventional tyrosine-dependent signaling pathway, a novel intracellular domain-dependent pathway, known as ligand and tyrosine kinase-independent (LYKi), has been recently described [130]. This domain spans residues 989 and 1276 in the β-subunit of the IR. A restricted number of mechanisms have been posited to explain the activation of LYKi signaling: firstly, the direct effects of IR in various compartments as it traverses the cell, and secondly, the interaction of the IR with other proteins in the cell in a kinase-independent manner [131,132]. A further mechanism involves the direct transcriptional control of the IR in the nucleus [133]. The precise activation of LYKi remains to be investigated. The activation of the LYKi pathway facilitates the IR to perform essential functions even in the absence of ligands, owing to the occurrence of multiple phosphorylation events. Nagao et al. [130] showed that this pathway is responsible for the upregulation of genes involved in extracellular matrix (ECM) organization and cholesterol biosynthesis, as well as the increased expression of proteins such as collagen and fibrillins. Moreover, it downregulates several immune/interferon-related genes and proteins, including those involved in cytokine, interferon, and JAK/STAT signaling. LYKi signals also augment the sensitivity to apoptosis through both intrinsic and extrinsic pathways. Additionally, it regulates the cell cycle and ATM signals and inhibits the cellular senescence phenotype, as well as the secretion of senescence-associated secretory phenotype (SASP).

#### 3.1.6. Activation and Signaling Pathway Differences between IR Isoforms

The signaling pathways activated by IR-A and IR-B are similar, but the relative activation of these pathways varies depending on the ligand bound, the specific isoform involved, and the target tissue. For example, in pancreatic β-cells, the type of IR to which insulin binds leads to the transcription activation of different target genes. The binding of insulin to IR-A promotes signaling through PI3K class Ia/p70s6k, resulting in insulin gene transcription, while binding to IR-B activates PI3K class II-like activity and PKB, leading to glucokinase gene transcription [134]. Furthermore, in neonatal hepatocytes expressing IR-B alone, there is more caspase-8 and caspase-3 induction, leading to subsequent apoptosis, whereas cells expressing IR-A alone stimulate the mitochondrial pathway of apoptosis. Interestingly, in the same cells, the co-expression of IR-A and IR-B protects cells from apoptosis [135]. In cells lacking IGF-1R but expressing IR-A, both insulin and IGF-II induce nuclear translocation of IRS-1, while in cells lacking IGF-1R but expressing IR-B, there is no IRS-1 nuclear translocation [136]. Moreover, the activation of a specific IR isoform results in different downstream effects by inducing various signaling pathways, depending on the ligand bound to it. For instance, IGF-2 promotes faster IR-B endocytosis, regulating its mitogenic action through endosomes, while insulin stimulation leaves IR-B at the cell membrane, enabling IR-B-dependent metabolic responses [137,138]. Although both insulin and IGF-2 induce the ERK1/2 and Akt pathways at similar peaks upon binding IR-A, the duration of stimulation differs. IGF-2 activation leads to a longer-lasting ERK1/2 pathway, whereas insulin prolongs Akt activation [49]. Similarly, studies have shown that IGF-2 is less effective than insulin in inducing the IRS/PI3K pathway but more effective in inducing the ShC/ERK pathway [47,139]. Additionally, IGF-2 binding to IR-A induces higher p70S6K:Akt and ERK1/2:Akt ratios than insulin, resulting in enhanced cell migration. On the other hand, insulin-induced activation of the PI3K/Akt pathway has more potent anti-apoptotic effects than IGF-2 [49,139]. In summary, while both IR-A and IR-B activate similar signaling pathways, their tissue distribution and affinity for insulin and IGF-2 can lead to different downstream effects in various cell types and physiological contexts.

#### 3.1.7. Intracellular IGFR Signal Transduction Network

The binding of IGFs to the α subunit of IGF-1R induces a conformational change in the β subunit, leading to the activation of RTK activity [140]. The β subunit contains multiple tyrosine residues that undergo phosphorylation upon ligand binding. This phosphorylation is facilitated by the tyrosine kinase of another β subunit within the tetrameric IGF-1R, a process known as autophosphorylation. Autophosphorylation further activates the tyrosine kinase of the β subunit. When the tyrosine 950 residue of the NPXY motif in the JM domain is phosphorylated, docking proteins like IRSs with the PTB domain can recognize this motif and bind to the β subunit, thereby becoming phosphorylated by the IGF-1R [141,142,143,144]. Phosphorylation of tyrosine residues, such as 1131, 1135, 1136, and 1221, activates receptor kinase, leading to cellular transformation and migration [145,146,147,148,149]. Additionally, phosphorylation of tyrosine 1250 and 1251 plays important roles in the internalization and degradation of the receptor [150,151].

Upon the binding of IGF-1 or IGF-2 to IGF-1R, the receptor becomes activated. Ligand-activated IGF-1R first binds to intracellular adaptor proteins, including Shc1 [152], Gab [153], and Crk [154] but predominantly IRS1 [155]. These adaptor proteins are crucial for transmitting downstream signals through the P13K-Akt1-mTOR pathway. When IGF-1R binds to IRS1, the latter interacts with the p85 regulatory subunit of P13K, initiating signals to Akt1 and mTOR. Activation of this pathway results in pleiotropic effects, including inactivation of the pro-apoptotic protein BAD [156,157,158,159,160]. Simultaneously, IGF-1R binds to Shc, which, in turn, interacts with the Grb2-SOS complex to activate the MAPK pathway [154]. Activation of the MAPK pathway leads to the transcription of several genes, such as Cyclin D, which drives cellular proliferation and differentiation. Cyclin D/Cyclin-dependent kinase 4 (CKD4) complex activation promotes E2F-dependent transcription, after liberating the E2F transcription factor from the retinoblastoma protein (Rb), facilitating G_1_/S-phase transition during cell-cycle progression [161,162,163]. Notably, the Akt pathway stabilizes cyclin D by acting on cell-cycle regulatory proteins and the inhibitory phosphorylation of glycogen synthase kinase-3 beta, p21, and p27 [164,165,166]. Moreover, it enhances cyclin D function by preventing the inhibition of the mammalian target of rapamycin complex 1 (mTORC1) by tuberous sclerosis complex (TSC) proteins, leading to ribosomal biogenesis and more efficient translation of cell-cycle progression-specific mRNAs essential for cellular growth and proliferation [167,168]. Furthermore, IGF-1R promotes cellular motility by activating IRS2, which, through poorly understood mechanisms involving the small G protein RHOA, focal adhesion kinase (FAK), and Rho-kinase (ROCK), influences integrin expression [156,157].

**Figure 4:**
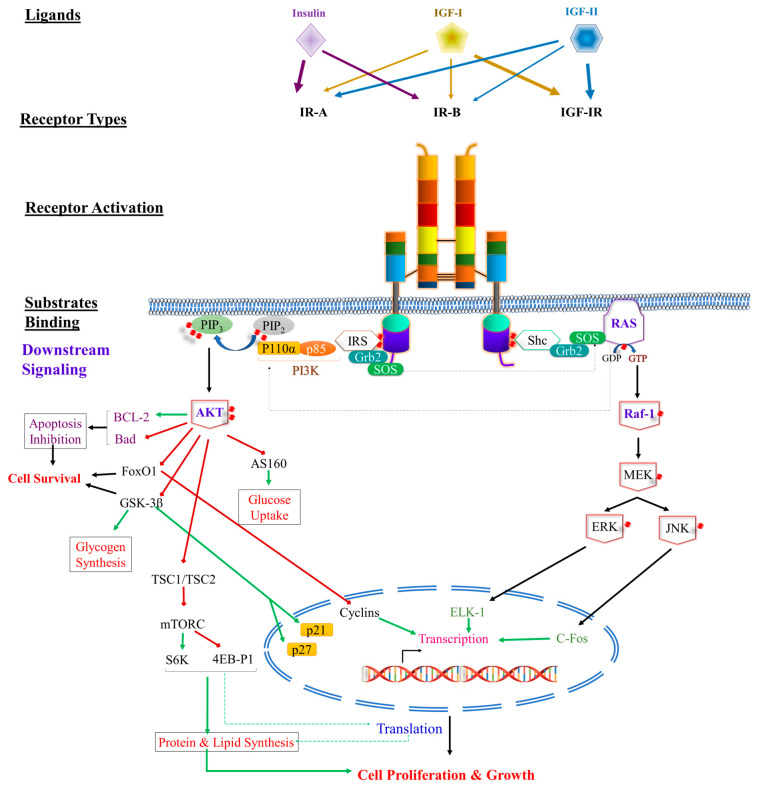
An illustration of the downstream pathways upon activation of IR and IGF-IR in physiological conditions. Specific differences between the receptors’ signaling pathways are explained in the text. Red circles are phosphorylation; green lines are excitatory signals; red lines are inhibitory signals.

As for IGF-2R, it acts as a repository for IGF-2 and does not possess intracellular signaling activity. Therefore, IGF-2R functions as a tumor suppressor gene. When IGF-2R loses its function, IGF-2 can bind to IGF-1R and promote tumorigenesis [158] (Figure 4). 

## 4. Role of IR and IGFR in Carcinogenesis

### 4.1. Role of IR Isoforms in Carcinogenesis

The IR plays a significant role in both metabolism and oncogenic effects, particularly in clinical scenarios characterized by compensatory hyperinsulinemia resulting from metabolic pathway resistance, such as obesity and diabetes. In many malignancies, IR is preferentially overexpressed as the IR-A isoform, as indicated in Table 2. The overexpression of IR-A is associated with various factors contributing to its carcinogenic effect. For instance, IR isoform overexpression may contribute to cancer cell stemness, tumor development, and resistance to targeted therapies by interacting with IGFs [169]. Local production of IGF-2 by epithelial and stromal cancer cells, along with an increased IR-A: IR-B ratio, supports the mitogenic response of cancer cells to insulin. The IGF-2/IR-A loop encourages cancer cell growth, migration, and self-renewal [90,169,170]. Moreover, IR regulates transcription factors involved in the epithelial–mesenchymal transition (EMT) process such as p53, Oct-4, and Nanog, promoting carcinogenesis and pluripotency. IR activation leads to the phosphorylation and inhibition of p53, which in turn inhibits both Oct-4 and Nanog, resulting in persistently activated Oct-4 and Nanog, ensuring cell survival [171,172,173].

In a study by Nowak-Sliwinska et al., it was revealed that IR-A is upregulated on the angiogenic vasculature in various human tumors, which correlates with poor prognostics and patients’ survival [190]. Similarly, Benabou et al. reported that an increased IR-A-to-IR-B ratio in hepatocellular carcinoma is associated with stem/progenitor cell features such as cytokeratin-19 and α-fetoprotein, as well as shorter patient survival after curative resection [191]. Interestingly, in the aforementioned study, it was the dysregulation of the IR-A-to-IR-B ratio rather than the upregulation of ligand expression that was commonly observed in cancers.

Recent discoveries have shown that the function and expression of IGF-1R and IR are significantly regulated by epithelial discoidin domain-containing receptor 1 (DDR1), a tyrosine kinase receptor for nonintegrin collagen crucial for embryonic development, extracellular matrix modification [192], and cell migration, survival, proliferation, and differentiation [193,194,195,196]. Dysregulated DDR function has been linked to the development of several human illnesses, including cancer, arthritis, and fibrosis. In the most aggressive cancers, IR-A is currently the most prevalent IR isoform expressed, and recent studies have indicated that DDR1 has the potential to modulate the IGF-2/IR-A loop [197]. The predominance of isoform A observed in cancer is attributed to abnormalities in both mRNA transcription and post-transcriptional processes, as shown in Table 3 [169]. 

Endothelial cells undergoing sprouting express a multifunctional proteoglycan known as decorin, which preferentially inhibits the physiologic responses of IR-A that are mediated by IGF-2 while leaving insulin- or proinsulin-dependent signaling unaffected. Decorin expression aids in capillary development and cell survival [222]. Therefore, depletion of decorin could impact the development and spread of tumors that rely on the IGF-2/IR-A autocrine loop [223]. 

In addition to surface receptors, several oncogenic fusion proteins arising from chromosomal translocations communicate with IRS adaptor proteins to promote tumor growth. Examples include the ETV6-NTRK3 gene fusion associated with pediatric spindle cell sarcomas and secretory breast cancer, the RET-PTC3 gene fusion linked to papillary thyroid cancer, and the NPM-ALK gene fusion linked to anaplastic large-cell lymphoma, which is a transforming oncogene [224,225,226]. The anaplastic lymphoma kinase (ALK) is present in neuroblastoma, pancreatic and breast cancer, and melanoma and has been found to be a rate-limiting factor for the development of glioblastoma, communicating through the IRS proteins [227]. 

### 4.2. Role of IGFRs in Cancer 

IGFRs play a critical role in development, growth, and cell survival, and their involvement in the pathogenesis of certain malignancies has been well established [228]. Increased expression of IGF-1R has been reported in various cancers, including Ewing sarcoma, breast cancer, prostate cancer, pancreatic cancer, melanoma, and other tumor types, contributing to faster tumor progression and, in some cases, poor prognosis [229,230,231]. Functional IGF-1R is essential for malignant transformation [232]. Interestingly, IGF-1R localization in the cytoplasm indicates receptor activation, while nuclear IGF-1R transmits tumorigenic signals [233,234,235]. Elevated expression of IGFRs has shown negative prognostic impacts (Table 4 and Table 5). In many cases, IGF-1R overexpression is a consequence of the loss of tumor suppressors, such as p53, breast cancer gene-1 (BRCA1), von Hippel–Lindau protein, and Wilms’s tumor suppressor WT1 [236,237,238]. However, it is the presence of ligands rather than receptor aberrations alone that drives IGF signaling in tumor cells [236]. Interestingly, low circulating IGF-1 concentration can protect against tumorigenesis [239,240]. Activated IGF-1R can also stabilize integrins and promote epithelial–mesenchymal transition (EMT), thereby facilitating cancer metastasis [241]. Uninhibited IGF/IGF-1R signaling can also result from decreased levels of insulin-like growth factor binding protein-3 (IGFBP-3), which competitively inhibits IGF/IGF-1R binding. Reduction in IGFBP-3 is often caused by proteolysis, and higher plasma proteolysis incidence has been observed in women with advanced stages of breast cancer [242,243]. Similarly, in prostate cancer, the tumor marker prostate-specific antigen (PSA) can cleave IGFBP-3 [244]. IGF-1R overexpression was shown also to be caused by NRF2 activation [245,246]. NRF2 is a well-known regulator of antioxidant response, as well as various metabolic and cellular functions, and has been identified as a key driver in the progression of cancer, metastasis, and chemoresistance. It has been demonstrated that NRF2 activation interacts with SP1, a potent transactivator of the IGF1R gene, thereby promoting the expression of IGF-1R and facilitating cancer progression. Interestingly, it has also been shown that oncogenic RAS signals can upregulate NRF2 [247], rendering the initiator of this vicious cycle questionable. 

Similarly, IGF-2R is associated with various malignancies with prognostic implications (Table 5). In the development of cancer, defects in IGF-2R are reportedly caused by loss of imprinting [248]. Given its role in clearing IGF-2, IGF-2R functions as a tumor suppressor, where the loss of its function can lead to an accumulation of IGF-2, promoting tumorigenesis. In fact, IGF-2R also regulates cell proliferation, apoptosis, migration, angiogenesis, and invasive ability [249]. Moreover, decreased IGF-2R induces lysosome dysfunction and inhibits autophagy [250,251]. The loss of monoallelic gene regulation renders the receptor susceptible to multiple mutations, resulting in an inactive gene copy and the absence of functional protein [252]. Additionally, genetic polymorphisms affecting IGF-2R clearance of IGF-2 ligands have been linked to a higher probability of developing oral, colon, and hepatocellular carcinoma [253,254,255]. Furthermore, several studies reported that IGF-2R knockdown suppressed the tumorigenic properties of tumors [256]. Though some studies reported increased expression of IGF-2R (Table 5), the contribution of IGF-2R overexpression to tumor development is still unclear. In cervical cancer, it was shown that upregulation of IGF-2R helped cells escape lysosomal-dysfunction-induced apoptosis via the transport of M6P-tagged cathepsins [251]. Similarly, in hemangiomas, high levels of IGF-2R were reported in a proliferative phase where the knockdown of IGF-2R significantly diminished the proliferative activity and induced apoptosis and cycle arrest with decreased expression of PCNA, Ki-67, Bcl-2, Cyclin D1, and E and increased the expression of Bax [257].

**Table 4:** Studies showing prognostic impact of overexpression of IGF-1R in various cancer types.

Cancer Type	Tissue Types/Cell Lines	Quantification Method	Causes of the Overexpression	Prognostic Implications	References
**Breast Cancer**	Breast tissue subtypes: Luminal type a and type b	Gene expression profiling (microarray)/Molecular profiling	Overexpression was hormonally driven	IGF-1R overexpression was predominantly seen in ER-positive (+) tumors contributing to chemoresistance, and reducing IGF-1R levels significantly reduced ER+ tumor size	[258,259,260]
	Triple-negative breast cancer		N/A	IGF-1R expression was found to be associated with a lower disease-free survival rate (*p* = 0.031).	[261]
**Colorectal Cancer**		Electrophoresis	IGF-1R has a relation to SNP implication in CRC	There was a significant association between IGF-1R rs2229765 polymorphism and advanced CRC (AA/AG vs. GG: OR = 3.06, *p* = 0.004)	[262]
**Esophageal Cancer**	Esophageal cells CE48T/VGH cell line	Northern blot analysis and ligand-binding assay	N/A	Overexpression of IGF-1R and autocrine growth regulation may concertedly control the proliferation of esophageal carcinoma	[263]
**Gastric Carcinoma (GC)**	N/A	Immunohistochemistry	N/A	IGF-1R overexpression positively correlated with MRP-1 overexpression (rp = 0.39, *p* < 0.01). IGF-1R and MPR-1 overexpression was correlated with poor prognosis of GC (*p* < 0.01)	[264]
**Head and Neck Squamous Cell Carcinomas (HNSCCs)**	Squamous cell	IHC	N/A	OS and DSS were reduced in patients whose tumors contained high membrane IGF-1R	[234]
**Lung Cancer**	Small-cell lung cancer (SCLC)	NA (serum and lung cancer histological tissues from small-cell lung carcinoma (SCLC))	N/A	IGF-1R overexpression leads to increased cell survival and suppressed cell apoptosis, whereas IGF1R silencing mediated by RNAi abrogates this response of NCI-H446	[265]
**Osteosarcoma**	Human primary and metastatic osteosarcomas	Reverse transcriptase polymerase chain reaction	N/A	_	[266]
**Prostate Cancer**	Malignant epithelia	IHC	N/A	High IGF-1R was associated with high risk of metastasis	[233]

DSS: Disease-specific survival; OS: Overall survival; DFS: Disease-free survival; SNP: Single-nucleotide polymorphism; MRP-1: Multidrug resistance-associated protein-1.

**Table 5:** Studies showing prognostic impact of altered expression of IGF-2R in various cancer types.

Cancer Type	Tissue Types/Cell Lines	Quantification Method	Expression	Causes of Altered Expression	Prognostic Implications	References
**Bladder Cancer**	Bladder carcinoma	qPCR and IHC	↓ in 70% vs. ↑ in 30%	N/A	Low expression is associated with poor prognosis. - Lower OS (*p* = 0.022) Worse clinicopathological features including higher histology grade (*p* = 0.001), higher tumor stage (*p* = 0.033), and LN metastasis (*p* < 0.001)	[267]
	Bladder carcinoma	PCR, FISH	↑	Autophagy-associated circular RNA hsa_circ_0007813 upregulation sponge hsa-miR-361-3p to regulate IGF-2R expression	Unfavorable prognosis	[268]
**Breast Cancer**	Triple-negative breast cancer	IHC	↑	N/A	Patients with IGF-2R-positive expression had lower OS (*p* < 0.001) and DFS rates than those with IGF-2R-negative expression (67.8% vs. 78.5%, *p* = 0.023)	[269]
**Cervical Cancer**	Cervical cancer tissue and cell lines	DNA microarray, IHC	↑	N/A	Poor prognosis Lower OS compared to cases with intermediate- and high-IGF-2R expression, *p* < 0.05 and *p* < 0.01, respectively, in Stage I	[251]
**Colon Cancer**	Adenocarcinoma malignant tissue	RNase protection assay	↑	N/A	_	[270]
**Liver Cancer**	Primary HCC	PCR	↓	LOH of M6P/IGF-2R	Poor Prognosis - OS (24.9% vs. 65.5%; *p* = 0.04) DFS (17.8% vs. 59.3%; *p* = 0.03)	[271]
**Lung Cancer**	NSCLC tissue and cell lines	IHC	↓ in 56% vs. ↑ in 44%	N/A	Low IGF-2R expressions had a poorer prognosis than those with high IGF-2R expressions - With ↓ expressions, PFS and OS are shorter (6.8 ± 2.1 vs. 8.8 ± 2.4, months, *p* < 0.001 and 11.1 ± 7.2 vs. 17.1 ± 2.8, months, *p* < 0.001) ↓ Expressions are associated with later tumor stage (*p* = 0.0013) and poorer differentiation status (*p* < 0.001)	[249]
**Oral Cancer**	Squamous cell carcinoma tissues from patients	PCR	↓	Gly1619Arg polymorphism of M6P/IGF-2R domain 11 (rs62989)	Loss of IGF-2R function increased risk of advanced stage of OSCC by 3-fold	[253]
**Osteosarcoma**	Various cell lines	Flow cytometry analysis	↑	N/A	N/A	[272]
**Pancreatic Cancer**	Islet cells, acinar cells, and ductal cells	ISH	↑	N/A	_	[273]

DSS: Disease-specific survival; ISH: In situ hybridization; PFS: Progression-free survival; OS: Overall survival; DFS: Disease-free survival; HCC: Hepatocellular carcinoma; LN: Lymph node; N/A: not applicable for the study.

## 5. Role of IR and Igfrs in Chemoresistance

### 5.1. Role of IR Isoforms in Chemoresistance

Numerous studies suggest that the overactivation of the IR-A pathway, induced by insulin and IGF-2, plays a direct and crucial role in cancer development and may contribute to resistance against various anti-cancer drugs. For instance, Heidegger et al. demonstrated the significant involvement of both IGF-1R and IR-A upregulation in prostate cancer carcinogenesis and its chemoresistance [274]. They also found that the downregulation of IR-A led to enhanced response to docetaxel and cycloheximide. Despite these findings, the exact mechanism underlying chemoresistance remains poorly understood.

Interestingly, the overactivation of the IGF-2/IR-A loop in cancer cells is a mechanism of adaptive resistance to anti-IGF-1R drugs [47,139,170,178,180,275]. Compensatory crosstalk between IGF-1R and IR has been observed, contributing to resistance against IGF-1R targeted therapies. Inactivation of IGF-1R pharmacologically has been shown to upregulate IR expression. Additionally, antitumor treatments may stimulate IGF-2 production, increasing drug resistance through IR-A binding. Consequently, IR can mediate primary resistance to IGF-1R target therapy, as IR overexpression leads to elevated levels of IR-A, which binds IGF-2 with high affinity, promoting tumor development. To combat this resistance, co-targeting IR and IGF-1R together has been proposed to enhance therapy efficacy and inhibit resistance against selective anti-IGF-1R drugs [276,277,278,279,280]. Dual inhibitors like OSI-906 have already been developed and tested in clinical trials [281]. Another approach involves targeting both IR and IGF-1R simultaneously using an artificial E3 ubiquitin ligase capable of recognizing specific proteins of interest. This engineered ubiquitin ligase comprises an IGF-1R/IR binding domain (the PTB domain of IRS-1) and a functional ubiquitin ligase domain, facilitating ubiquitin-mediated proteolysis. This strategy has potential benefits as cancer cells often overexpress multiple oncoproteins, making it challenging to overcome survival pathways with single-target therapies [282,283]. Therefore, dual inhibitors targeting both IR and IGF-1R hold promise in inhibiting tumor progression effectively without impairing IR’s normal function [274].

### 5.2. Role of IGFRs in Chemoresistance

IGFRs play a significant role in contributing to chemoresistance by counteracting the effect of anti-cancerous medications. One way IGFRs promote chemoresistance is through their involvement in DNA repair, thereby preventing the full effects of anti-cancer drugs that target DNA damage from taking place [284]. Additionally, IGFRs can render anti-epidermal growth factor receptor (EGFR) treatments ineffective. EGFR is a tyrosine kinase receptor that drives cellular proliferation, growth, and survival through downstream signaling pathways, and it is often upregulated in cancers [285]. While anti-EGFR therapies consist of neutralizing monoclonal antibodies and selective tyrosine kinase inhibitors that inhibit oncogenesis [286,287], IGFRs interact with these antibodies, allowing the continuous action of the EGFR/HER2 kinase family signaling pathways. Furthermore, IGFRs can hinder the effectiveness of drugs targeting increased estrogen in ER-positive breast cancer, promoting chemoresistance [288,289]. In breast cancer, IGF-1R has also been shown to resist the effect of trastuzumab, an anti-EGFR/HER2 antibody, by inhibiting the SRC/FAK/FoxM1 signaling pathway [290]. The overexpression of IGFR receptors can impact the used inhibitors in clinical therapy [291]. This aspect must be considered when devising strategies to combat the effects of IGFR in order to improve the clinical outcomes of chemotherapy [291,292,293]. Consequently, many medications have been developed as therapeutic agents targeting IGFR, as its increased levels are considered a challenge in cancer patients. For example, the anti-insulin-like growth factor 1 receptor antibody EM164, a monoclonal antibody, can be used as a therapeutic agent in childhood neuroblastoma [294]. It is noteworthy that the expression of IGF-1R in chemoresistant cells exhibits a pulsatile pattern [295]. Specifically, it is observed to be overexpressed upon initiation of chemotherapy; however, its level subsequently diminishes as cancer cells attain complete resistance. A study by Dhave et al. [296] showed that transcriptional regulators like Runt-related transcription factor 1 (RUNX1) and Forkhead Box O3 (FOXO3a) collaboratively drive the dynamic modulation of IGF-1R expression by binding the IGF1R promoter, producing a transcriptional surge during the onset of resistance. In the same study, it was demonstrated that upon attaining complete resistance, cellular co-operation between RUNX and FOXO3 ceases, leading to a reduction in IGF-1R expression due to the diminished negative feedback signals originating from AKT-FOXO3a. 

Compared to IGF-1R, the role of IGF-2R in chemoresistance is poorly studied. However, a study by Tian et al. [249] showed that low IGF-2R levels in non-small-cell lung cancer (NSCLC) contribute to poor response to cisplatin. Similarly, Sun et al. showed that IGF-2R inhibition by Trop2 contributed to NSCLC resistance to gefitinib [297]. Conversely, Takeda et al. [251] demonstrated that upregulated IGF-2R in cervical cancer is associated with poor response to cisplatin. 

## 6. Challenges of Quantification Methodologies and Future Insights

### 6.1. Quantification of IR Isoforms

Currently, the evaluation of IR isoform mRNA expression is primarily conducted at the tissue level, and more recently, advancements have allowed for assessment at the cellular resolution in situ. Flannery et al. developed a highly specific qRT-PCR assay to quantitatively measure the levels of human IR isoforms and IGF-1R on the same scale [298]. The assay involved the design of human-specific primers for IR-B, IR-A, and IGFR RT-PCR, targeting IR and IGFR sequences. Common reverse primer annealing with exon 12 is used along with isoform-specific forward primers that anneal across exon–exon junctions on each respective isoform. For IGFR primers, the aim is to target regions of low IR homology. Another technique, single-cell RNA sequencing (RNA-Seq), aims to identify the presence of IR isoforms in single cells. However, RNA-Seq remains technically challenging due to the low levels of IR mRNA expression and the minimal base-pair differences between the two isoforms [299]. It is important to note that mRNA expression may not accurately reflect the actual protein levels in cells and tissues [63]. 

While Western blotting (WB) and immunostaining are commonly used to assess IR protein levels, attempts to develop isoform-specific antibodies have been challenging. This difficulty is likely due to the small difference between the two isoforms and the fact that the 12 differential amino acids reside in a poorly accessible area of the receptor [300]. In addition, WB and immunohistochemistry have certain limitations, including being both semi-quantitative with moderate sensitivity, and their results rely on antibodies that may not possess sufficient specificity.

### 6.2. Quantification of IGFR 

IGF-1R and IGF-2R levels in tissue samples are commonly measured using methods like WB and IHC. Ligand-binding assays, such as ELISAs and radioimmunoassays, can also be employed to determine the concentration of IGFRs in biological fluids, including IGF-1R and IGF-2R. However, it is important to note that these techniques may be influenced by staining variability, tissue heterogeneity, non-specific binding, or interferences from other substances in the sample, such as circulating ligands or binding proteins, which can lead to inaccurate results. Flow cytometry has been utilized to measure IGFR levels on the cell surface and subsequent intracellular signaling in various cancer types, such as Ewing’s sarcoma, osteosarcoma, and breast cancer [301,302,303]. Flow cytometry is a fluorescence-based immunoassay, and its results can be affected by factors such as non-specific binding, cell autofluorescence, and antibody cross-reactivity. Another common method is IHC, which enables visualizing IGFRs in tissue samples. 

### 6.3. Future Insights

Recent advancements in super-resolution imaging techniques, such as single-molecule localization microscopy (SMLM) [304] and stimulated emission depletion microscopy (STED), offer the potential for high-resolution imaging of IGFRs in living cells and tissues. In SMLM, the target protein is labeled with a fluorescent dye, with the labeling process varying depending on the specific SMLM imaging technique [305]. However, these methods are still in the early stages of development and validation, and there are doubts about their ability to quantify IR isoforms using antibodies.

Visualizing IR isoforms in live cells would be intriguing as it could provide a better understanding of ligand binding to the IR isoforms at the cell membrane. One potential approach is to use small molecules like aptamers, which can differentially bind to the IR isoforms, conjugated with fluorescent dyes to visualize endogenous IR receptors in living cells. This could provide insights into intracellular dynamics during health and disease. Aptamers have demonstrated the ability to block IR-mediated signaling in an isoform-specific manner in vitro [306], showing their potential for IR isoform detection. 

Mass-spectrometry-based methods, such as multiple reaction monitoring (MRM) and parallel reaction monitoring (PRM), as well as targeted proteomics, also hold promise for quantifying IR isoforms. Liquid chromatography-tandem mass spectrometry (LC-MS) can ionize and analyze analytes of interest based on their mass/charge ratio, offering greater specificity, linearity, reproducibility, and lower limits of quantification compared to conventional methods [307]. Mass spectrometry overcomes some of the antibody specificity issues associated with techniques like WB and ELISA, as it measures unique peptides from each protein, enabling absolute quantification rather than just relative quantification. While these methods require specialized equipment and expertise, they have been successfully used to develop various assays for protein isoforms [308]. 

## 7. Conclusions

In spite of extensive research, the exact mechanism by which both IR isoforms and IGFRs contribute to carcinogenesis and chemoresistance remains poorly understood. This review aims to explore the intricate roles of IR isoforms and IGFRs within the insulin and IGF signaling (IIS) pathway. The study delves into the extensive network of ligands, receptors, and binding proteins that together orchestrate a multitude of functions, with implications for diverse processes such as carcinogenesis and chemoresistance.

A detailed genetic analysis of IR and IGFR structures has revealed a fascinating array of isoforms arising from alternative splicing, each exhibiting distinct affinities for ligands. The complexity of this system is underscored by the connection between overexpression of the IR-A isoform and critical factors such as cancer stemness, tumor development, and resistance to targeted therapies. Similarly, heightened expression of IGFRs accelerates tumor progression and chemoresistance, thereby underscoring the critical role they play in disease pathology.

This review not only highlights the intricate interplay between IRs and IGFRs but also elucidates their contributions to resistance against chemotherapeutic and anti-IGFR drugs as well as the prognostic implications of these receptors in various tumors. In light of this, we propose the potential for a more effective therapeutic strategy by concurrently targeting both receptors to overcome the challenges posed by chemoresistance.

However, a significant hurdle to understanding how the dysregulation of these receptors contributes to carcinogenesis and chemoresistance is the currently used quantification methods. With the emerging technological advancements, it is hoped that many obscurities will be unraveled.

## Figures and Tables

**Table 1:** Differences between the IRs, IGF-1R, and HR receptors.

Characteristics	IR-A	IR-B	IGF-1R	IR-A/IGF-1R	IR-B/IGFR	IGF-2R
**Ligand-binding affinity**						
Pro-insulin	4.5 ± 0.6 [49]	31.0 ± 6.3 [49]	>100 [49]	_	_	
Insulin	0.4 [50] − 2.7 [51]	0.49 [50] − 2.6 [51]	>30 [52] − >1000 [50]	1.1 [53] − 342 [54]	1.1 [53] − 325 [54]	
IGF-I	>30 [52]	>30 [52]	0.2 [52] − 1.49 [55]	0.01 [53] − 6 [54]	0.01 [53] − 12 [54]	
IGF-II	3.3 [47] − 15 [56]	36.0 ± 3.8 [47]	0.6 [52] − 13 [10]	0.18 [53] − 0.7 [57]	0.19 − 15 [53]	1.87 [58]
**Distinctive structure**	Absence of 12 AA	Presence of 12 AA encoded by Exon 11	Formed by 21 exons	_	_	Lacks tyrosine kinase
**Function**	Embryogenesis and fetal development	Regulates glucose production and renal function of glomeruli and tubules. Because of its higher tyrosine kinase activity (2-folds), it is more involved in metabolic signaling [28]	Mediates apoptosis-inhibiting signals, and enhances cell metabolism and protein synthesis as well as tumor transformation and malignant cell survival			IGF-II scavenging, as well as regulating and trafficking lysosomal enzymes from the Golgi apparatus to lysosomes
**Distribution in cells**	More in brain cells, cancer cells, human placenta, osteoblasts precursor, spleen cells, and skeletal muscle	More in liver cells, mature osteoblasts, epididymal adipose tissue, kidney cells, and thyroid	Ubiquitous so it can be found in the kidney, ovary, and prostate			Ubiquitous and thus can be found in the liver, kidney, lung, adipose tissues, skeletal muscle, and placenta

**Table 2:** IR isoform expression in various cancer types.

**Hormone-dependent cancer**							
**Cancer**	**Cell type**	**IR**	**IR-A**	**IR-B**	**IR-A:IR-B ratio**	**IGFR**	**References**
Breast	h-BC	↑	↑	↓	↑	_	[174,175,176]
	h-BC	↑	↑	↓	↑	↓	[175]
Endometrial Adenocarcinoma		_	↑	↑	↑	↑	[177]
Ovarian	h-OV cell lines	_	↑	↑	_	_	[178]
Prostate (Androgen-Dependent)	h-PC	↑	_	_	_	_	[179]
	h-PC	↑	↑	↑	_	_	[180]
	h-PC	↑	_	_	↑	↓	[181]
	LNCaP and VCaP	↑	↑	↓	_	_	[182]
**Hormone-independent cancer**							
**Cancer**	**Cell type**	**IR**	**IR-A**	**IR-B**	**IR-A:IR-B ratio**	**IGFR**	**References**
Bladder	h-BLC specimens	↑	↑	_	_	_	[183]
Colon	m-PCA, h-CC	↓	_	↓	_	_	[184]
Liver	h-HCC	↑	↑	↓	↑	_	[185]
	r-HCC	↑	_	_	_	↑	[186]
	m-HCC	↑	↑	_	↑	↓	[187]
Lung	h-NSCLC	_	↑	↓	↑	_	[188]
Osteosarcoma	h-OS	_	↑	_	_	↑	[189]
Prostate (Androgen-Independent)	DU145	_	↓	↑	↓	_	[182]
	PC3	_	↑	↓	↑	_	[182]
Thyroid	h-TC	_	↑	_	_	↑	[170]

↑: increased; ↓: decreased; _: no available information.

**Table 3:** Summary of mechanisms of dysregulated IR expression in cancer.

Alteration of Transcription				
IR Dysregulated Expression Molecular Pathways	Function	Dysregulation Consequences	Cancer Type	References
Sp1, HMGA1, FOXO1	IR transcription gene	IR upregulation	Breast, gastric, ovarian, liver, colorectal, prostate	[169,198,199]
p53 inactivation	Tumor suppression gene	IR upregulation		[25]
**Post-Transcriptional Dysregulation Due to Alternative Splicing Regulatory Factor and Non-Coding RNAs**				
**(A) Alternative Splicing Regulatory factor**				
**I. CUG-BP1 increase**	Splice silencer Facilitates exon 11 exclusion Monitors the translation	Increased IRA:IRB ratio since it favors IRA expression	Breast, lung, colorectal	[22,25,200,201,202,203]
**II. hRNP H increase**	Interferes with CUG-BP1 to inhibit the splice of IR exon 11 Involved in pre-mRNA splicing, exporting mRNA, mRNA stability, and mRNA translation Engages in an interaction with CUG-BP1 that maximizes the inhibition of IR exon 11 inclusion	Increased IRA:IRB ratio		[25,204,205,206]
hRNP A1	- Represses the inclusion of exon 11 by attaching to the intron 11 5′ splice site	Increased IRA:IRB ratio	NSCLC	[24,207]
2. hRNP A2/B1	- Is a nuclear RNA-binding protein responsible for mRNA splicing	Increased IRA:IRB ratio	Multiple myeloma, NSCLC, PDAC, HCC, breast	[208,209,210,211]
**III. Loss of SRSF3 (** **Serine/arginine-rich protein 3)** **and SRp20**	Alternative splicing regulatory proteins that promote exon inclusion Also regulate mRNA export and translation SRSF3 with SRp20 promotes exon inclusion at exon 11	Loss of SRSF3 and SRp20 causes increased IRA:IRB ratio since favoring IRA expression	HCC	[212,213,214]
**IV. Muscleblind-like (MBNL) protein downregulation**	Splicing enhancers of pre-mRNA Counteracts CUG BP1’s effects Involved in exon 11 inclusion by engaging with HnRNP H Favors IR-B isoform	When downregulated, IR-B levels decrease, increasing IRA:IRB ratio	Breast, HCC, lung, renal	[25,215,216]
**V. mir-128 downregulation**	Post-transcriptional modulation of gene expression	Increased IRA:IRB ratio	Breast, melanoma, ALL	[217,218]
**VI. mir-15b/16-2 downregulation**	- Primarily through base pairing to 3′-untranslated sections (UTRs), causes destruction of mRNA transcripts and/or repression of translation	Increased IRA:IRB ratio	Lymphocytic leukemia	[219]
**VII. mir-1 downregulation**	Its deletion can affect cyclin D2 and IGF-R; thus, it controls the apoptosis of β cells	Increased IRA:IRB ratio	Bladder cancer	[220]
**(B) Non-Coding RNAs**				
Enhanced IRES-mediated IR mRNA translocation to the ribosomes	Initiates translocation	IR upregulation		[221]
